# Dural tears with cauda equina herniation following percutaneous endoscopic lumbar discectomy: a case report and literature review

**DOI:** 10.3389/fsurg.2024.1487567

**Published:** 2024-10-15

**Authors:** Shiwei Xie, Mingwei Luo, Heng Xiao

**Affiliations:** Department of Orthopaedics, Panzhihua Central Hospital, Panzhihua, Sichuan, China

**Keywords:** percutaneous endoscopic lumbar discectomy (PELD), complication, dural tear, lumbar disc herniation, case report

## Abstract

Lumbar disc herniation (LDH) is a prevalent condition that severely impacts patients' quality of life and work capacity. Traditional surgical treatments like laminectomy, while effective, involve significant invasiveness and potential complications, including long-term spinal instability and recurrent symptoms. With the advancement of minimally invasive techniques, percutaneous endoscopic lumbar discectomy (PELD) has become a popular option due to its reduced trauma and faster recovery. However, PELD, while beneficial, carries risks, including complications that may not be immediately evident. This report presents the case of a 60-year-old female patient who underwent PELD for L4/5 disc herniation but experienced significant postoperative complications, including increased pain and neurological symptoms. Initial conservative management failed, and further investigations suggested possible postoperative infection, though this was later ruled out through surgical exploration and bacterial cultures. The patient subsequently underwent open surgical exploration, which revealed extensive tissue damage and required additional interventions, including a minimally invasive lateral anterior approach for stabilization and fusion (MIS-OLIF). Postoperative recovery was successful, with complete symptom resolution and stable spine alignment at a six-month follow-up. This case highlights the complexity of managing PELD-related complications and underscores the importance of thorough diagnostic evaluation and the potential need for additional surgical interventions to ensure long-term patient outcomes.

## Introduction

Lumbar disc herniation (LDH) is a common condition that significantly affects patients' quality of life and ability to work. In severe cases, it can lead to incapacitation, imposing a substantial social burden. Although traditional laminectomy is effective, it requires general anesthesia and extensive dissection of posterior muscles and ligaments. This approach often damages the spinal venous plexus, leading to significant blood loss, prolonged surgery, and extended hospitalization. Furthermore, long-term spinal instability may develop, with approximately 10% of patients experiencing a recurrence of clinical symptoms. The advancement of minimally invasive spinal surgery has led to the adoption of percutaneous endoscopic lumbar discectomy (PELD) for treating lumbar disc herniation. PELD offers several advantages, including reduced trauma, faster recovery, and precise outcomes. However, the success of PELD is highly dependent on the surgeon's experience. Despite these benefits, PELD is associated with various complications, some of which may not be immediately apparent during the procedure (1). In severe cases, these complications can result in neurological disorders. This report discusses a patient admitted to the Department of Orthopaedics at Panzhihua Central Hospital, highlighting the clinical diagnosis and treatment of PELD-related complications. The case serves as a reference for understanding the clinical manifestations of these complications. Informed consent for this study was obtained from the patient.

## Case description

A female patient is a 60-year-old farmer who is usually in good health. The patient had no significant medical history or family history of spinal disorders. She had not reported any relevant psychosocial factors or genetic predispositions that could have influenced her condition. There was no history of prior interventions related to lumbar disc herniation before the current episode of care. To ensure patient confidentiality, all identifying information has been de-identified in accordance with institutional and journal requirements. The patient was diagnosed with “L4/5 disc herniation” in a tertiary hospital 1 month ago due to “low back pain for 3 years and pain and numbness in the left lower limb for 1 month”, and underwent percutaneous endoscopic L4/5 discectomy and decompression under local anesthesia, and the patient's low back pain and pain and numbness in the left lower limb were significantly relieved after the operation. After the operation, the patient experienced significant relief from low back pain and left lower extremity pain and numbness. Unfortunately, 2 weeks after the operation, the patient's lumbosacral and left hip pain increased, and the pain was aggravated by walking and changing position, and relieved by resting in the supine position. Three weeks after surgery, the patient's lumbosacral pain symptoms worsened and her right hip was painful. Her condition did not improve significantly after “local block” treatment at a hospital, and she was then admitted to our hospital for treatment. Three days after admission, the patient gradually developed numbness in the left thigh.

**Figure 1:**
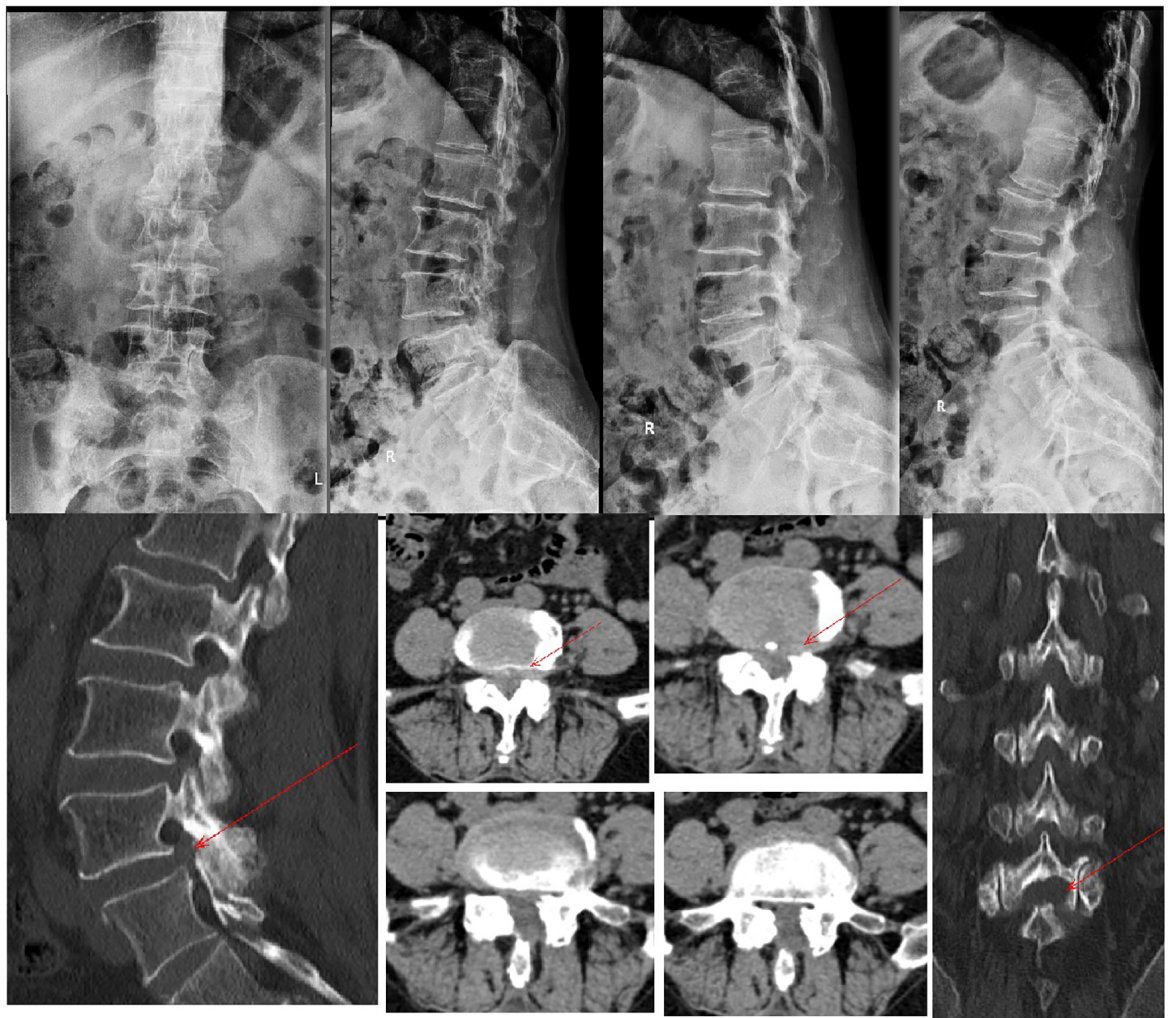
X-ray suggests L4/5 intervertebral space narrowing. Lumbar spine power position x-ray suggests good stability. CT scan of the lumbar spine suggesting lumbar disc herniation with partial loss of the left lamina of lumbar 4 (red arrow).

**Figure 2:**
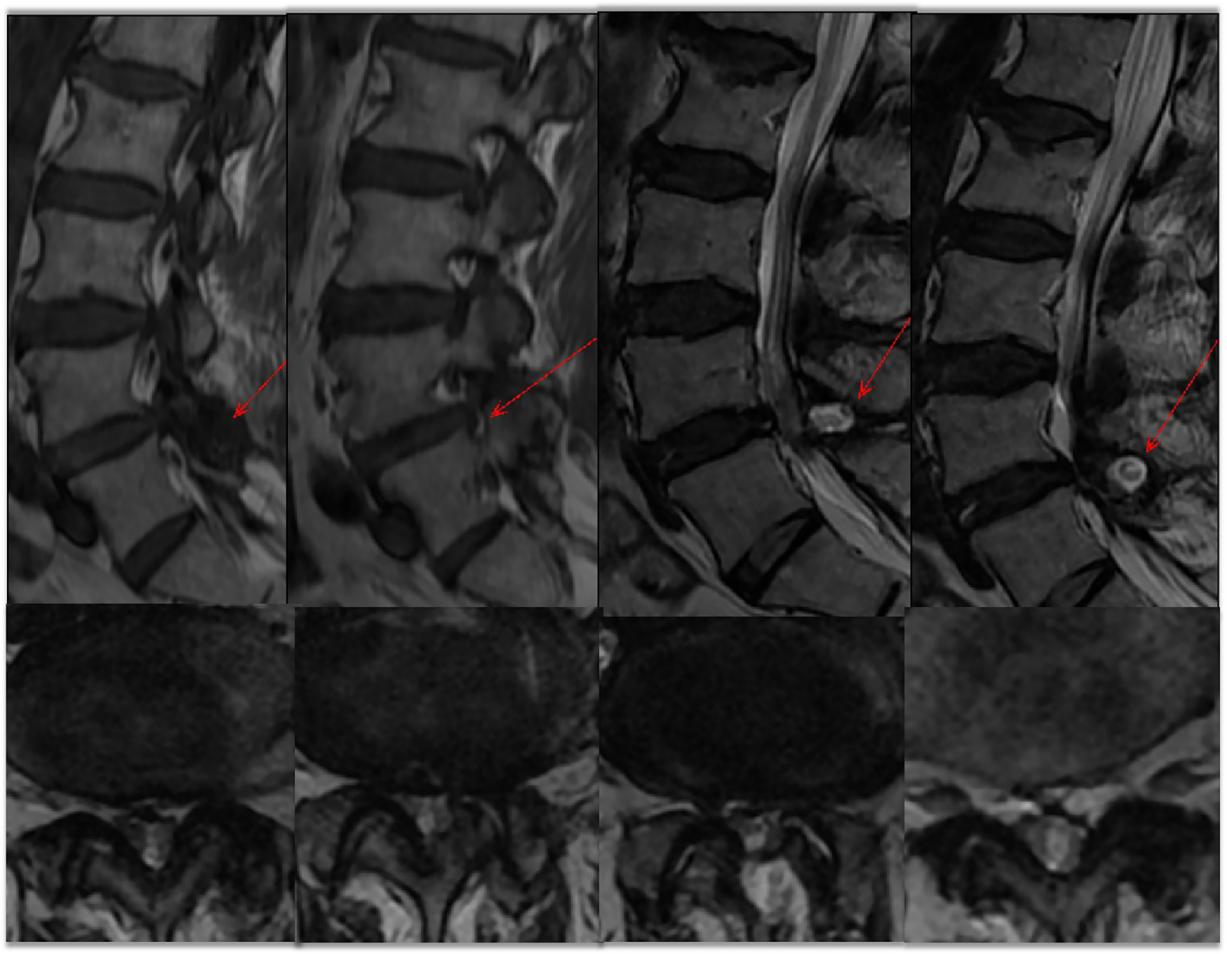
MRI of the lumbar spine showed a lumbar 4/5 disc herniation. A “fish-eye sign” is seen posterior to the spinal canal (red arrow). Cross-section suggests spinal stenosis.

Upon admission, we performed a comprehensive physical examination of the patient, who had limited lumbar flexion and extension, lower lumbar tenderness, deep buttock tenderness, and a positive left calf straight leg raising test and strengthening test (50°).The sensation and muscle strength of both lower limbs were not abnormal. X-ray and CT scan of the lumbar spine was shown in Figure 1. The L4/5 intervertebral space was narrowed, but with good stability. CT scan was shown the left side of the L4 vertebral plate was missing, the L4/5 commissural space was blurred and hyperplastic, and the L4/5 intervertebral disc was bulging and protruding to the left posterior side. MRI image was shown in Figure 2: the flat cystic lesion in the L4/5 intervertebral space was pushing the dural sac forward slightly, and the L4/5 intervertebral disc was protruding to the left side. The results of routine blood tests were as follows: neutrophil ratio 83.20% ↑, erythrocyte sedimentation rate (ESR) and C-reactive protein were normal (CRP).

## Diagnosis

The patient was a middle-aged woman. Her symptoms recurred postoperatively to a severe degree. The pain mainly affected the lumbosacral region and bilateral buttocks. This pain could be suddenly aggravated after walking and changing position, and could be relieved after bed rest. Taking into account the patient's history, physical examination, and ancillary tests, we initially thought that she had postoperative recurrence of PELD, but considering that the blood counts suggested a high neutrophil ratio and the lumbar MRI showed a cystic lesion at the posterior margin of L4/5, the possibility of postoperative intervertebral space infection could not be completely ruled out, although the likelihood was very low.

## Treatment

**Figure 3:**
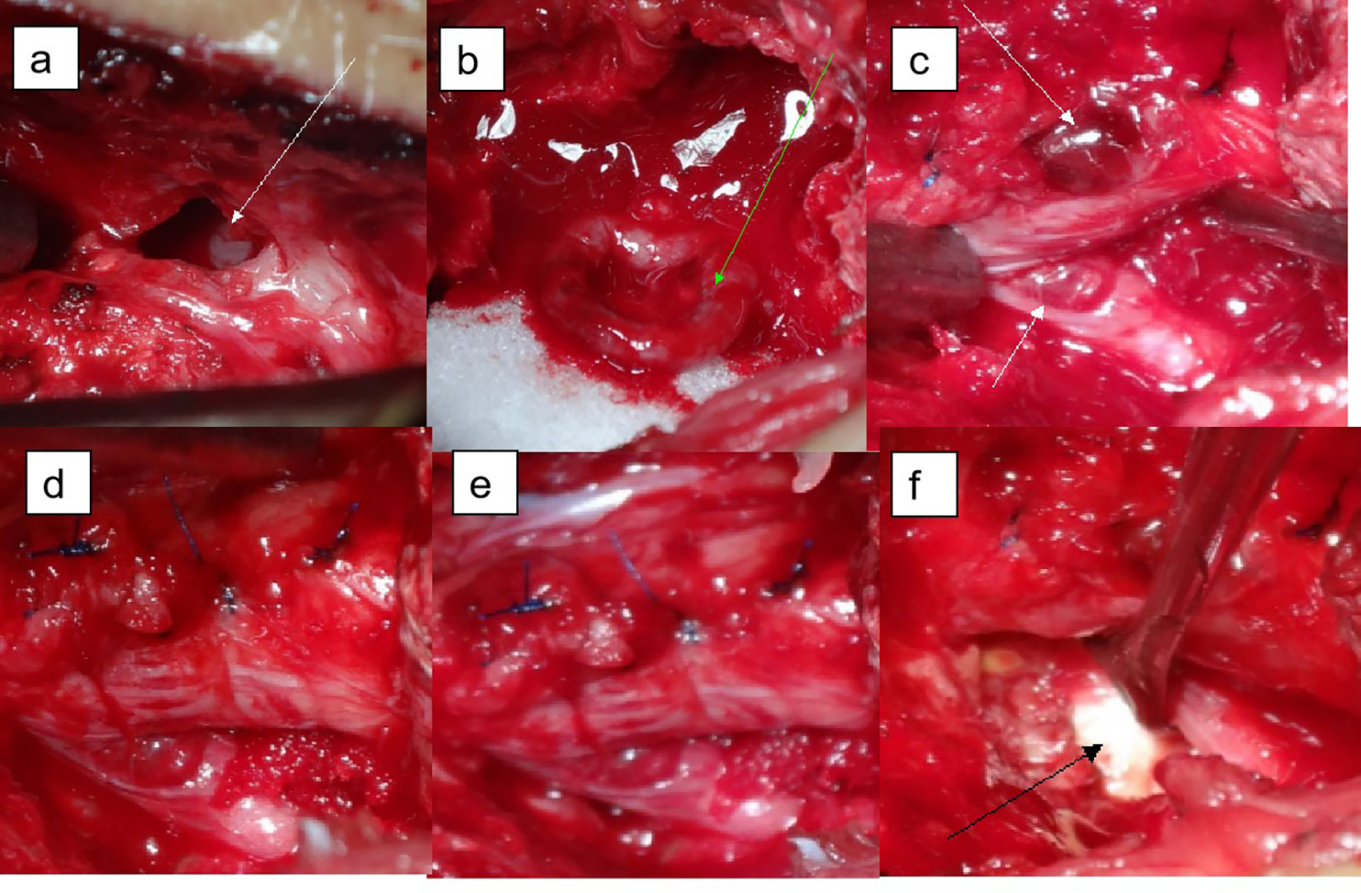
Visualization during operation. **(a)** The left portion of the lamina of lumbar 4 is absent, and white cords can be seen in the spinal canal (white arrows); **(b)** after L4 laminectomy, a dural tear with nerve root herniation was found; **(c)** part of the dura mater was repaired; the nerve root was compressed and bruised; **(d)** complete repair of the dura mater, the bilateral nerve roots were found, and the axillary injury and congestion of the left nerve root of L5 were found (white arrows); **(e)** the right L5 nerve root is intact compared to the left, and the nerve root sheath is complete and smooth; **(f)** the L4/5 disc was herniated (black arrow), a discectomy was performed.

To clarify the diagnosis and provide symptom relief, the patient initially underwent bilateral small joint closure at the L4/5 level. However, the procedure did not result in any symptom improvement. Consequently, open surgical exploration was performed. Intraoperatively, we observed edema, dark redness, and partial inactivation of the muscles in the operated area, as well as a laminar defect on the left side of L4/5. Upon incision of the lamina, a white streaky material was identified within the spinal canal, consistent with a dural tear and cauda equina herniation (Figure 3). Retraction of the cauda equina allowed us to perform a successful dura repair. During the procedure, we resected part of the small joints bilaterally to gain access and thoroughly explore the nerve root. The left nerve root was found to be ventrally damaged and edematous, and a herniated disc fragment was observed protruding towards the left posterior. The disc was resected, and further exploration revealed laxity of the nerve root. A drainage tube was placed in the operative area, and because we could not entirely exclude the possibility of infection preoperatively, we collected soft tissue samples for bacterial culture without performing a 1-stage fixation. Postoperatively, the patient's pain was significantly relieved. Bacterial culture results from the drainage fluid and soft tissues were negative for infection, ruling out intervertebral space infection. Given that the entire L4 lamina and part of the bilateral articular process were removed, resulting in spinal instability, we decided that a fixation and fusion procedure was necessary. Due to the patient's prior history of two spinal surgeries and significant localized adhesions, a posterior approach posed high risks. Therefore, we opted for a minimally invasive lateral anterior approach (MIS-OLIF) for L4/5 discectomy, followed by intervertebral CAGE-assisted fusion and internal fixation. This approach minimized trauma while providing the necessary stability to the spinal column.

## Outcome and follow-up

**Figure 4:**
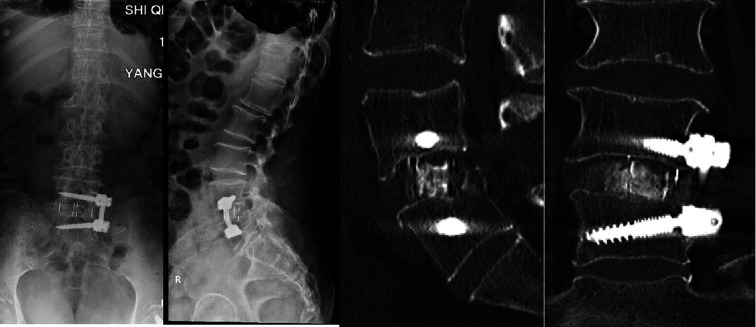
X-ray and CT scan of lumbar spine after six months show that lumbar spine has good force line, good nail bar position and good support. CT showed partial intervertebral fusion.

One week after surgery, the thoracolumbar spine structure was stabilized, the low back pain was completely relieved, and at the six-month follow-up, the pain did not recur, and the spine was well supported by the line of force, the position of the nail rods, and the cage support, and there was a possibility of fusion at L4/5.To restore the stability of the patient's spine, we chose MIS-OLIF to stabilize and balance the middle column before reconstruction (Figure 4), and the patient's symptoms were completely relieved.

## Discussion

Lumbar disc herniation (LDH) arises when disc material, such as the nucleus pulposus or annulus, protrudes beyond the normal margins of the disc, compressing the spinal nerve roots (2). This condition is a prevalent cause of low back pain and sciatica, and it frequently presents in spine surgery with a high incidence rate. Although physiotherapy, accupunture, and chiropractic management are effective treatment strategy (3), 20% of patients require hospitalization for surgery (4). LDH is a common cause of low back pain and sciatica. It is a common and frequently encountered condition in spine surgery and has a high incidence. Nearly 70%–85% of people experience at least one episode of back pain with or without leg pain during their lifetime. The social burden is high, and approximately 10%–20% of patients require hospitalization for surgery. The procedure for PELD was first introduced by Kambin (5) in 1992 and gradually developed into an option for the treatment of LDH. Therefore, PELD treatment of lumbar disc herniation has been accepted by more and more people and is widely used (6–8). However, due to the limitations of endoscopic procedures and substantia lanatomica requirements, longer learning time is required. If the indications for surgery are not well understood and the surgical techniques are not mastered, various complications may occur, such as recurrence, nerve root injury, dural tear, dysesthesia, discitis, headache, hematoma, visceral injury, and wound infection (9). Ruan W, et al. (1) conducted a meta-analysis and found that compared with the incidence of complications and fenestration in laminectomy and nucleus pulposus removal, the incidence of complications in PELD was 4.69% and the incidence of vertebral fenestration was 2.33%. Although the difference was not statistically significant, it suggested that the former procedure had a higher incidence of complications than the latter. In addition, complications are common in procedures performed by spine surgeons using minimally invasive treatment techniques. Postoperative recurrence is a common complication of PELD. There was no statistically significant difference between postoperative recurrence and open surgery (10). Some patients with recurrence can achieve complete remission with conservative treatment, but surgery is required when conservative treatment is ineffective. Dural tear is a common surgical complication in patients undergoing lumbar surgery, and its incidence is approximately 1.8%-17.4% (11). C. I. Ju et al. (12) reported a study of narrative analysis, comparing the complications of the transforaminal and interlaminar approaches. They found that dural tears are overwhelmingly common (2.19%) in interlaminar decompression. M. Pan et al. (13) reported that the incidence of dural tear increased to 1.1% when percutaneous endoscopic lumbar discectomy (PELD) was switched from an “inside-out” technique to an “outside-in” technique. Dural injury by instruments or radiofrequency, spinal canal adhesions, large disc fragments, and a loose dura are risk factors for dural tears. There are several main reasons for spinal dural tears caused by UBE spine endoscopic surgery. (1) Beginners easily make mistakes because the visual field under endoscopy is a 2-dimensional plane and is easily blurred. (2) UBE does not require retraction of the anatomical structure to expose the dura mater, which is quite different from other techniques. (3) Patients with complex conditions require operations of long duration, increasing the risk of spinal membrane tears. (4) During the operation, the injected saline squeezed both sides of the dura mater, causing the area to fold. The central area may be damaged during ligamentum flavum resection. (5) When using high-speed drills, the peripheral fibrous bands and vascular bundles of the dura may stretch around the drill neck, causing larger tears (9). Dura tear is the most frequently reported complication of endoscopic surgery in works of various literatures. Since nerve root herniation causes serious symptoms and secondary nerve damage, it is important to prevent nerve root herniation in dura defects. Until now, the gold standard treatment for dural damage has been open dural repair. However, recently, sealing dura defects using absorbable hemostatic materials (such as collagen fleece) has been widely performed in endoscopic surgery, eliminating the need for open surgery and general anesthesia. The initial landing should be as close to the target as possible, and a complete herniotomy after thorough release of the ring anchorage is a key to success and may prevent complications (14). However, there are no clear statistics on the incidence of dural rupture associated with PELD procedure, and there are few reports on this subject. Because of the difference from open surgery, PELD dural tears are more difficult to detect, which is related to the fact that PELD is filled with saline and maintains a high water pressure. Small dural tears are difficult to detect, and small dural tears do not necessarily cause clinical symptoms during or after surgery. However, as time passes and position changes, intradural pressure increases. A small number of symptomatic patients may experience back pain or numbness in the lower extremities, and sometimes an urge to urinate. If intracranial pressure is reduced, patients may experience headache or neck stiffness, and even some patients may experience severe neurological dysfunction. Refractory root pain occurs because there is not enough space to accommodate the leaked CSF. The nature of this type of pain is uncertain and it manifests as electric shock-like pain that tends to increase with change of position or walking and is adequately relieved in certain positions. With open lumbar surgery, there have been reports of nerve root fluid leakage due to dural tears (11, 15, 16). There are few reports of neural crests in patients with dural tears after PELD, but the clinical manifestations are not typical because the imaging findings are unclear and the diagnosis is difficult.

The symptoms of low back pain in the patient treated in our department and reported here are not typical. The initial pain was mainly low back pain, and the pain worsened when the position was changed. The VAS score was 8, and the pain was relieved by lying down. The clinical features of the patient, combined with the auxiliary examination and lumbar CT examination, revealed bilateral L5/S1 articular process hyperplasia, and the low back pain was aggravated by activity or a change in body position, so we suspected it was due to small joint disorders and osteoarthritis. Therefore, the patient underwent bilateral facet joint closure surgery under C-arm fluoroscopic guidance. After the operation, the symptoms of low back pain were not relieved, and bilateral buttock and left thigh pain gradually appeared. The entire department discussed the case again and decided to perform an open surgical exploration. The results confirmed a dural tear at the cauda equina. This complication is extremely rare, although the incidence of dural tears is high, but there are few symptoms and few nerve root spasms. Although it is easier to diagnose after symptoms appear, it is easily misdiagnosed or missed. Removal of the nerve root can cause severe neurological dysfunction, which should be given due consideration by the spine surgeon. In this case, preoperative MRI showed a “fish-eye” cystic mixed signal at the posterior margin of the L4/5 spinal canal. This signal can be one of the characteristic imaging findings of nerve root spasm following dural tear. It can be used as a reference for spine surgeons. This imaging phenomenon does not necessarily occur when the dural tear is accompanied by nerve root spasm. Therefore, the clinical diagnosis should be based on the characteristics of the patient's history, symptoms and signs, and imaging examination to avoid misdiagnosis.

In the absence of a definitive diagnosis, surgical exploration carries some risk. During the exploration, in order to fully expose the area, the nerve roots are decompressed and the bilateral part of the facet joints and disc tissue is removed, destabilizing the spine. When the postoperative culture results were all negative, the second phase of fusion fixation was performed. When choosing a fixation method, we discussed a variety of possibilities considering the patient's history of two posterior approach surgeries, a large amount of localized scar adhesion, and a limited surgical field. Therefore, we chose the anterior OLIF procedure. The OLIF procedure is performed through an anterior and posterior retroperitoneal approach, and the surgery can be performed below the passageway with less trauma and bleeding. The surgical approach provides mechanical stability and implant space for the lumbar spine, increases the contact area between the implant and the bony endplate, increases the fusion rate, has better biomechanical strength, and can correct the lordotic deformity of the lumbar spine. It is a safe and effective spinal fusion technique under neuroelectrophysiological monitoring. In a prospective study of 15 patients with lumbar spondylolisthesis, Sardhara Jayesh et al. (17) found that the OLIF procedure for L2/L5 segment fusion achieved good results in terms of fusion rate and speed of recovery. In a case study of adult spinal deformity, Anand N, et al. (18) pointed out that OLIF is believed to definitely improve the sagittal and coronal balance of the spine with a lower incidence of perioperative and postoperative complications.

This case highlights the complexity of managing complications associated with PELD, particularly the rare occurrence of dural tears with cauda equina herniation. Our stepwise approach, from conservative management to open surgical exploration and ultimately stabilization via a minimally invasive lateral anterior approach, underscores the importance of adapting treatment based on the evolving clinical picture. The OLIF technique, which offers advantages such as reduced trauma and enhanced spinal stability, proved effective in this case, demonstrating its utility when posterior surgical approaches pose significant risks. While the case provides valuable insights, it is limited by its single-case nature and lack of long-term follow-up data, which may restrict generalizability. Existing literature has documented the risks associated with PELD, but reports of cauda equina herniation remain exceedingly rare. By adding to the body of knowledge on PELD-related complications, this case supports the necessity of prompt diagnosis and individualized intervention to achieve favorable outcomes. Future studies involving larger cohorts and extended follow-up will be crucial in validating the long-term efficacy of OLIF in similar complex cases.

## Conclusion

PELD has demonstrated advantages in the treatment of lumbar spine disorders, including lumbar disc herniation, but the surgical learning curve is steep, and less experienced spine surgeons should approach this technique with caution. Surgeons should tailor treatment choices to the individual patient's condition to ensure the most effective and appropriate treatment. Complications of recurrence after PELD are not uncommon. However, a dural tear with nerve root herniation is a rare complication, and the diagnosis is difficult and should be given sufficient attention by spine surgeons. The “fisheye sign” may be a characteristic image of dural tear with nerve root hernia, but more cases are needed to support this theory, which can provide some reference for clinicians.

## Patient perspective

The whole repair process took some time. Although I had to go through several surgeries, I am very satisfied with the complete resolution of my pain problem.

## Data Availability

The original contributions presented in the study are included in the article/Supplementary Material, further inquiries can be directed to the corresponding author.
